# Antiseptic Treatment of Diseases of the Dental Pulp

**Published:** 1883-11

**Authors:** 

**Affiliations:** Liege, Belgium


### ANTISEPTIC TREATMENT OF DISEASES OF THE DENTAL PULP.

BY DR. ROSENTHAL OF LIEGE, BELGIUM.

READ BEFORE THE AMERICAN DENTAL SOCIETY OF EUROPE, AT COLOGNE.

Before I give you my own conception of antiseptic treatment of the diseases of the dental pulp, allow me to present some of the ideas given by Witzel in his work on this subject.

In the first chapter, entitled, General Communications on the Treatment of the Dental Pulp, he says: "When pulps are freshly exposed and have not yet been in contact with saliva, I generally succeed in saving them ; but when they are inflamed the same treatment does not give me such good results."

In the second chapter, under the name, Action of Arsenious Acid, he says that when a tooth cannot be filled, he prefers to extract rather than to devitalize it. However, when the patient wishes to keep the tooth he thinks it may be his duty to do this. The deep action of arsenious acid is not demonstrated. "I have shown," he says, "at the meeting at Cassel, specimens demonstrating that arsenic applied to a healthy, uninjured pulp, attacks only the superficial tissue of the organ. The same thing occurs in inflamed pulps." One of the specimens was a molar exposed at one of the cornua, where the action of the medicament was shown at the point of the exposure, and in the direction of the softened tissues.

Though the action of arsenic is not entirely known, it is undoubted that its special action is the cause of great changes at the apex of the roots. The circulation of the blood continues even after the sensibility has disappeared in the cauterized parts, and stops only sometime afterward. You may find that in the parenchyma of the pulp, blood extravasated from the vessels which have probably been ruptured by the medicament.

Arsenic has no action whatever on the dentine, and to prove this Witzel makes three experiments. In one he applies arsenic with oil of cloves on a section of a tooth, and does not find any change. In the second he applies it with creosote, and a day afterwards finds a little spot stained, but in applying creosote alone the same spot reappears. The third experiment was to cut a healthy temporary canine into two parts, one of which he left for eight days in arsenious paste. In comparing the two portions under the microscope he does not find the least action on the dentine.

Chapter third treats of the question of capping. The following are the chief points he makes.

The exposed part of the pulp must be entirely cleared from any decomposed tissue.

The ingredients used must be such as to favor the return to normal conditions.

The exposurê must neither be cauterized or irritated by the cap.

The covering must be in close contact with the pulp, and be strong enough to resist the pressure needed to condense an amalgam filling.

As a general rule, a tooth whose pulp is exposed should never be immediately filled with gold.

He then gives his mode of procedure, which consists in washing the cavity with a mixture of phenol and tannin, while he excavates all the decayed tissues with spoon-shaped excavators. Should the pulp be exposed and bleed, he uses the same mixture, which he leaves some minutes on the pulp. Then he covers the exposure with a varnish made of collodion, gutta percha and phenol, and covers with oxy-chloride of zinc, and fills with amalgam. If any pus should be found after excavating, he thinks that the part of the pulp in the crown ought to be amputated.

In the fourth chapter he says that it is difficult to judge at first whether the pulp should be capped immediately or cauterized and amputated. He only amputates pulps of molars, and sometimes those of the bicuspids, when the pulp chamber has a suitable shape.

When a non-exposed pulp has given pain for a couple of hours at a time, and after it has been exposed it is found of a cherry red color and inflamed, he thinks it should be amputated.

Before removing the dressing and the arsenic he cleans the gum around the tooth, and disinfects it. Then he prepares the cavity in such a way that the pulp may be easily reached, both by the sight and by the instruments. When all is ready he takes a sharp drill, dips it in phenol, rapidly removes the rest of the dentine which covers the pulp, cuts the pulp at the base, and bathes it in phenol. He then caps it in the same manner as the ordinary exposed pulp.

To conclude, we may say that Mr. Witzel's work is most commendable from the studies of the action of arsenious acid, by which he has been led to the discovery of this mode of treatment. The illustrations are remarkably good, and add a great deal to the value of the book.

This treatment I have employed for about eighteen months, and have received much satisfaction from it. The weak point of it is when he applies phenol to a simple non-inflamed pulp, and it is, I think, the reason of the failures of which he complains. A pulp so exposed and non-inflamed, even if it has given pain, ought to be considered as a simple cut in any other part of the body, and all our efforts should tend to have the wound healed by first intention, and as there is no infection there should be no escharotic nor antiseptic medication. Generally, pulps in such condition may be recognized. The principal symptom is when all pain stops after the cavity has been thoroughly excavated and is free of all material which could excite the pain. Sometimes, however, slight pain may continue on account of the air coming in contact with the pulp, but we may easily allay it by applying a little piece of dry wool-cotton on the exposure.

Another point in which I cannot agree with Mr. Witzel is when he advises amputation without trying any other means to preserve the integrity of the pulp. I think the example he gives turns entirely against his theory, when he says that a dentist who has to pronounce his diagnosis on a diseased pulp is in the same situation as a physician who, having to examine a patient, would put him in a barrel and look at him through the bung-hole. When a pulp does not stop aching after the thorough excavation of the decayed tissue, it may surely be considered as partially inflamed. Here we have infection, and with reason we may use escharotics and antiseptics. After disenfecting I treat the exposure as usual, but add a little excess of phenol. If the pain does not stop, then only would I advise the treatment recommended by Witzel, and amputate the pulp.

I have but few words to add. In science, classification is of the greatest importance for comprehension. Formerly we used to classify caries under eight varieties: the white, the black, the dry, etc.; to-day we have only three: caries of the first, the second, and the third degree.

In medicine we have the same classification for many diseases; for example, burns are divided into those of the first, the second and third degree.

Why should not we classify the diseases of the pulp in the same manner, and say when a pulp is exposed and not inflamed, that it is of the first degree; when it is partly inflamed it is of the second degree; and when it is entirely engorged that is of the third degree. A single word would thus make us understand to what class of diseased pulps we are referring.

